# Does the 6-minute walk test in hospitalized COPD patients exclusively correlate with lung function parameters or should psychological factors also be taken into account?

**DOI:** 10.1371/journal.pone.0232587

**Published:** 2020-05-04

**Authors:** Michèle Borgmann, Martina Ivanda, Yalda Hadizamani, Markus Mohaupt, Robert Bals, Rudolf Lucas, Jürg Hamacher, Volker Köllner

**Affiliations:** 1 Internal Medicine and Pneumology, Lindenhofspital, Bern, Switzerland; 2 Lungen- und Atmungsstiftung Bern, Bern, Switzerland; 3 MediClin Rose Clinic, Horn-Bad Meinberg, Germany; 4 Internal Medicine, Sonnenhofspital Bern, Bern, Switzerland; 5 Internal Medicine V—Pneumology, Allergology, Respiratory and Environmental Medicine, Faculty of Medicine, Saarland University, Homburg/Saar, Germany; 6 Division of Pulmonary Medicine, Vascular Biology Center, Medical College of Georgia, Georgia Regents University, Augusta, GA, United States of America; 7 Department of Behavioral Therapy and Psychosomatic Medicine, Rehabilitation Center Seehof, Federal German Pension Agency, Teltow, Germany; 8 Department of Psychosomatic Medicine, Psychosomatic Rehabilitation Research Group, Center for Internal Medicine and Dermatology, Charité—Universitätsmedizin, Berlin, Germany; University of Lausanne, SWITZERLAND

## Abstract

The 6-minute walk test is generally considered a standard test for the evaluation of short-term maximal physical performance. It has not been evaluated whether psychological factors, such as anxiety or depression, affect the performance or the results of the test. The main aim of this study was to investigate whether a correlation exists between psychological factors and the data from the 6-minute walking test. The study cohort consisted of 85 (♀ = 34 and ♂ = 51) 66 ± 10 (mean ± SD) year-old patients with chronic obstructive pulmonary disease (COPD) hospitalized for disease exacerbation. Forced Expiratory Volume in the first second (FEV_1_) (% predicted) as predictor for lung function, as well as anxiety and depression symptoms assessed using the Hospital Anxiety and Depression Scale (HADS) as psychological predictors were collected. Bivariate correlations and hierarchical linear regression models were used to analyse the correlations. Walking distance was on average 260m ± 107m and ranged from 64m to 480m. HADS was negatively correlated with 6-min walking distance (r = 0.441, p = .0009, r = -.523, p = 00006). Hierarchical linear regression showed that FEV_1_ alone explained 33%, and together with the psychological variables anxiety and depression explained 42% of the variance of results from the 6-minute walking test. These findings demonstrated that 11% of the data correlated with the psychological variables alone (*p* = .011). The effect size for lung function (*f*^2^ = .717) and psychological variables (*f*^2^ = .352) were high, whereas the socio-demographic variables sex, age, educational level and BMI could not explain any additional variance in our cohort. In conclusion, our study indicates that psychological factors such as symptoms of depression and anxiety are associated with lower physical functional performance in the 6-minute walking test. As such, these factors should also be assessed. Future research is needed to show if treatments of anxiety and depression can improve the walking distance in COPD patients.

## Introduction

The 6-minute walking test is a submaximal exercise test for assessing physical endurance, including main cardiovascular, pulmonary and neuro-musculoskeletal performance. The aim of the test is to evaluate exercise performance including the effect of medical interventions such as rehabilitation, cardiac bypass interventions, pulmonary lung volume reduction surgery, or drug treatment by walking a maximal distance in 6 minutes [1, 2]. Due to its simplicity, the test is closely related to daily life activities. Its broad applicability allows for the inclusion of frail and elderly patients. As such, the 6-minute walking test mainly reflects physical performance for everyday activities. Consequently, results from the test are often used as a clinical outcome parameter or as a biomarker associated with disease burden and all-cause mortality [3–8]. Although most frequently used in COPD patients, the test can also be applied to patients with interstitial lung disease, pulmonary hypertension and heart failure, as well as in neurologic patients.

COPD patients have a lower 6-minute walking distance than healthy individuals [9], which correlates with their morbidity and mortality [10–13]. A 6-minute walking distance below 350 meters or a decline of 30 m or more over 1 year was associated with significantly higher mortality in COPD patients [10, 14, 15]. In long-term, prospectively studied cohorts, the 6-minute walking distance in patients with severe COPD continued to decrease over time, while FEV_1_ [16] changed much less or not at all [17]. As such, key lung functional parameters such as FEV_1_ or the ratio of residual volume to total lung capacity (RV/TLC) have limitations as predictors for the course of COPD. This suggests that the 6-minute walking test can be considered as a reliable prognostic indicator to predict mortality and morbidity in COPD patients but that, apart from lung function parameters, also other measurements should be taken into account [18, 19].

Several factors including actual physical activity and exercise amount [20], sex [21], body composition [22], severity of dyspnea [23], extent of emphysema [24] and exacerbation history [25] were proposed to influence the 6-minute walking test in COPD patients. Besides deconditioning, more advanced systemic involvement may influence the test, as worsening apoptosis in muscle biopsies [26] and increased levels of markers of oxidative stress and inflammation were described in more severe COPD patients [27]. Moreover, increased comorbidity was demonstrated to add to the 6-minute walking distance decline over time [17]. The focus of these studies was mainly on demographic and somatic factors. A study by Ingle et al., 2006 including a cohort of 571 patients with heart failure and 688 control patients demonstrated for the first time that, in addition to demographic and somatic predictors, also psychological predictors such as self-reported anxiety and depression can be associated with the 6-minute walking distance [28]. The authors assessed anxiety and depression by 6-item Likert scales, but not by standardized tools. Therefore, a limitation of their study was that no robust conclusions on the influence of psychological factors on 6-minute walking distance could be drawn. Another study with healthy volunteers showed correlations between the 6-minute walking distance and the psychological component of quality of life [29].

The identification of psychological predictors has potential for the promotion of improved functional performance. While demographic characteristics cannot be influenced and somatic characteristics are difficult to influence, anxiety and depression can be mitigated through available treatments. Based on the findings described above, the aim of the present study was to assess the relationship between the 6-minute walking test and anxiety and depression symptoms, using for the first time standardized measurements in COPD patients. The results of the study suggest that, in addition to the somatic component of physical functioning, also predictive psychological variables should also be taken into account in the 6-minute walk test in patients with COPD.

## Materials and methods

### Sample

The study involved 85 (♀ = 34 and ♂ = 51) 66 ± 10 (mean ± SD) year-old COPD patients devoid of other important comorbidities that were mainly hospitalised for either a COPD exacerbation or pneumonia, and that had a 6-min walk assessment before discharge. Only patients assumed as having as principal and unique disease of clinical importance COPD were included. Patients with “important comorbidity”, including severe heart failure or severe rheumatologic disease were excluded from the study. This setting is the most frequent setting of performing 6-minute walk test in COPD patients worldwide. The studied patient cohort in this psychosomatic study in COPD patients included all hospitalised COPD patients [30]. All patients fulfilled at least the criteria for GOLD stage II (defined by FEV1 ≥ 50% but < 80%), due to the fact that there were only COPD patients requiring hospitalisation, which virtually eliminated mild disease situations. 33 patients were assigned to GOLD stage II (classified as "moderate"), 25 to GOLD stage III (classified as “severe” and defined by FEV1 ≥ 30% but < 50%), and 27 to GOLD stage IV (classified as “very severe”, with FEV1 < 30%) [31]. The study was approved by the Ethics Committee (KEK-No. 003/11) of the University of Bern in Switzerland. All patients were above the age of 18 years, with ages ranging from 41 to 83 years. The majority of the patients had finished elementary school. The consent to participate was by verbal consent given to the investigator (T.L.) and the date range was from 2007–2010.

### Measuring instruments

The 6-minute walk test allows physical performance to be evaluated by measuring the distance walked on level terrain over a certain timeframe and was conducted according to a standardized protocol using the guidelines of the American Thoracic Society [3, 32].

As a lung function predictor, FEV_1_, the volume exhaled in one second during a forced expiration started at the level of total lung capacity (TLC) was measured by spirometry. As psychological predictor, the anxiety and depression symptoms were measured by the Hospital Anxiety and Depression Scale (HADS). HADS is the most widely used questionnaire internationally for assessing these symptoms, especially in patients with somatic diseases. The subscales HADS-anxiety and HADS-depression range is from 0 to 21. Values below 7 are normal, values from 8 to 10 are marginal, values from 11 to 14 indicate severe symptoms and values from 15 to 21 indicate very severe symptoms [33, 34].

In addition, sociodemographic data (age, gender, educational level, measured from 1 = no graduation to 5 = university degree), the body mass index (BMI calculated as weight indicated in kg /(height)^2^ where height is indicated in m), the “body-mass index, airflow obstruction, dyspnea and exercise capacity index in chronic pulmonary disease” (BODE index) and the modified British Medical Research Council dyspnea index (mMRC) were recorded. The BODE index is a multidimensional prognostic 10-point scale growing beyond lung function, where a higher result is associated with a higher risk of death from COPD [35]. The mMRC is a classification of dyspnea for COPD patients, which ranges from 0–4. The higher the degree, the more severe the shortness of breath [36].

### Statistical analyses

Means and standard deviations for all normally distributed variables and the median and percentile for variables that are categorically or not normally distributed were computed for the whole sample and also separately for the three GOLD stages. To identify correlations between the 6-minute walk test and the somatic (BMI, FEV1, BODE Index, mMRC) and psychological (HADS-depression, HADS-anxiety) variables, product-moment correlations according to Pearson were calculated. In addition, the predictors for the 6-minute walk test were determined using hierarchical linear regressions.

In addition, a hierarchical linear regression was performed to analyse whether the psychological factors, in addition to the lung function variable FEV_1_, can explain part of the variance of the 6-minute walk test, whereby the lung function FEV_1_ was included in the first block and the psychological predictors, HADS-anxiety and HADS-depression, in the second block. Sociodemographic variables were included in a third block. Furthermore, using the backward selection the independent explanatory value of the HADS subscales on the 6-minute walking distance was enabled to determine.

Data analysis was done using IBM SPSS Statistics 24.0. The critical value for statistical significance was defined as p < .05* [37]. The standardized regression coefficients can be interpreted as effect variables following the correlation coefficients. According to Cohen, β ≥ .10 corresponds to a weak effect, β ≥ .30 to a moderate effect and β ≥ .50 to a strong effect [38]. In addition, the effect sizes *f*^2^ were calculated according to Cohen (1992)[39], which can be interpreted as follows: *f*^2^ ≥ .02 weak effect, *f*^2^ ≥ .15 moderate effect and *f*^2^ ≥ .35 strong effect.

## Results

### Descriptive statistics of somatic and pulmonary function variables

The investigated patients had an average BMI of 25.7 kg/m^2^ ± 5.9 kg/m^2^. FEV_1_ was 44% ± 19%. The examined patients reached on average 260 ± 107 meter, with the shortest distance 64 meters and the longest 480 meters (median = 276m). The median of the BODE index was 3.0 (P10 = 0.6, P90 = 5.2) (0 = best value to 10 = worst value) and the one of the modified Medical Research Council degree of dyspnea was 2.0 (P10 = 0, P90 = 3) (0 = dyspnea during heavy exertion up to 4 = dyspnea during dressing/undressing). Table 1 shows the results of the somatic and pulmonary function as well as the demographic variables (gender and age) for the three GOLD stages.

**Table 1:** Means, standard deviations or median and percentile of the demographic variables, somatic, pulmonary function and HADS variables of the 85 hospitalized COPD patients without major comorbidities separated for the three GOLD stages.

	GOLD II (*n* = 33)	GOLD III (*n* = 25)	GOLD IV (*n* = 27)
Gender	11 female, 22 male	7 female, 18 male	16 female, 11 male
Age (years)	68.7 ± 8.5	66.8 ± 11.0	60.8 ±8.0
BMI (kg/m^2)^	27.7 ± 6.6	25.7 ± 5.5	25.8 ± 5.8
FEV_1_ (%)	64.6 ± 8.5	40.0 ± 6.5	22.0 ±4.8
6-min-distance (m)	355 ± 58	254 ± 93	206 ± 103
BODE index	3.0 (*P*_10_ = 0.6, *P*_90_ = 5.2)	5.0 (*P*_10_ = 3, *P*_90_ = 9)	7.0 (*P*_10_ = 5, *P*_90_ = 9)
mMRC	2.0 (*P*_10_ = 0, *P*_90_ = 3)	2.0 (*P*_10_ = 1, *P*_90_ = 4)	3.0 (*P*_10_ = 2, *P*_90_ = 4)
HADS-anxiety	5.7±4.4	8.4±3.9	10.2±4.4
HADS-depression	5.5±4.4	9.1±4.8	9.6±5.0

BMI: Body mass index; FEV_1_: Forced Expiratory Volume in the first second, BODE index: body-mass index, airflow obstruction, dyspnoea and exercise capacity index; mMRC: Modified British Medical Research Council; HADS: Hospital Anxiety and Depression Scale; GOLD II (FEV_1_ ≥ 50% but < 80%), GOLD III (FEV_1_ ≥ 30% but < 50%), GOLD IV (FEV_1_ < 30%); n: number of patients.

### Descriptive statistics of psychological variables

The mean of HADS-anxiety was 8.0 ± 4.6 and of HADS-depression 7.9 ± 5.0. Overall, 46.2% of participants had a value higher than 7 on the anxiety and depression scale. In more detail, in HADS-anxiety 43 persons (53.8%) showed normal (inconspicuous), 10 persons (12.6%) marginal and 27 persons (33.6%) conspicuous values, which indicates severe to very severe symptoms. A similar pattern is found for HADS-depression: 43 persons (53.8%) have normal (inconspicuous), 11 persons (13.8%) marginal and 26 persons (32.4%) conspicuous values, which also indicates severe to very severe symptoms. The two subscales show high reliability with a Cronbach's alpha of α = .81 (HADS-anxiety) and α = .87 (HADS-depression). Table 1 shows the means and standard deviations of the HADS subscales for the three GOLD stages. Table 2 demonstrates that no further important relationships between variables could be observed.

**Table 2:** Correlation matrix of 6-minute walking distance with lung function variables and HADS subscales for the 85 hospitalised COPD patients without major comorbidities.

	age	sex	BMI	FEV_1_	mMRC	BODE index $	Education	HADS anxiety	HADS depression
6 min walking distance	.087	.135	.218	.600***	-.673***	[-.795] ^$^	-.253	-.441***	-.523***
Age		.239*	.082	.359***	-.055	-.112	.107	-.180	-.133
sex			.327**	.194	-.210	-.171	.215*	.096	.036
BMI				.242*	-.193	-.379**	.001	-.017	.065
FEV_1_					-.639***	-.741***	-.133	-.358***	-.401***
mMRC dyspnea						.708***	.066	.489***	.455***
BODE Index $							.148	.350**	.344**
Education								.124	.248**
HADS-anxiety									.569***

*p < .05

**p < .01

***p < .001

BMI: Body mass index; FEV_1_: Forced Expiratory Volume in the first second, BODE index: body-mass index, airflow obstruction, dyspnoea and exercise capacity index; mMRC: Modified British Medical Research Council; HADS: Hospital Anxiety and Depression Scale.

^$^ Note that BODE index contents categories of 6 min walking distance, of FEV_1_, mMRC, and BMI. Therefore the BODE index is *per se* auto-correlated with 6 min walk, with FEV_1_, with BMI, and with mMRC. As a consequence, the value has to be interpreted in this context.

### Correlation between psychological variables and the 6-minute walk test

HADS-anxiety (r = -.44, *p* = .0009) and HADS-depression (r = -.52, *p* = .00006) showed moderate negative correlations with the 6-minute walking test: The stronger the anxiety and depression symptoms in a patient, the shorter the walking distance within 6 minutes. Further correlations with demographic and pulmonary function variables are shown in Table 2.

The hierarchical linear regression showed that FEV_1_ and the psychological variables HADS-anxiety, and HADS-depression together accounted for 42% of the variance of the 6-minute walk test, with 11% of these related to the psychological variables alone (*p* = .011). These findings are emphasized by the high effect size for lung function (*f*^2^ = .717) and psychological variables (*f*^2^ = .352). Socio-demographic variables (sex, age, and educational level) and the BMI could not explain any additional variance of the 6-minute walk test. The regression coefficients and the explained variance for each block are shown in Table 3.

**Table 3:** Model summary of the hierarchical linear regression analyses with three blocks (lung function, psychological variables and sociodemographic variables) for the 85 hospitalized COPD patients without major comorbidities.

		Adjusted R-Squared	R-Square Change	Coefficient	*F*	*p*
Lung function		.33	.34		25.96	.00003
	FEV_1_			β = .43***		.001
Psychological Variables		.42	.11		13.29	.013
	HADS-Anxiety			β = -.08		.550
	HADS-Depression			β = -.32*		.029
Sociodemographic Variables		.38	.03		5.13	.717
	Sex			β = .04		.766
	Age			β = -.16		.259
	Educational level			β = -.08		.546
	BMI			β = -.03		.841

*p < .05

**p < .01

***p < .001

BMI: Body mass index; FEV_1_: Forced Expiratory Volume in the first second; HADS: Hospital Anxiety and Depression Scale

The backward selection showed that FEV_1_ (*β* = .439, *p* = .0004) and HADS-depression (*β* = -.360, *p* = .003) alone explained 42% of the variance. Therefore, HADS-anxiety did not have an additional contribution (*β* = -.077, *p* = .597).

## Discussion

The main finding of our study using for the first time quantitative standardised methods is that in addition to the lung function component (FEV_1_), also psychological factors, mainly the depressive symptoms assessed with the HADS, correlate with results from the 6-minute walk test in patients with COPD. Indeed, nearly a quarter of the explained variance of the regression model, which included FEV_1_ and HADS subscales, can be attributed to depressive symptoms, and negatively affect the 6-minute walking distance.

It has been shown several times that depressive symptoms often occur as a comorbidity with COPD [40–43][44]. Upon surgical lung volume reduction in COPD patients, an important association was found between lung function improvement and amelioration of psychological factors [45] including a virtual normalization of HADS scores (not shown). Two existing meta-analyses report a prevalence rate of 24.6% and 27.1% in COPD patients with comorbid depression [41, 42]. Patients with severe COPD have an about 2.5-times higher risk of developing depression than control groups [46]. The prevalence rate of depressive symptoms in our cohort was 46% and therefore significantly higher than the prevalence rates found in literature. However, this may be due to the fact that our cohort exclusively consisted of hospitalised patients. However, depression prevalence or the prevalence of depressive symptoms likely also depend on the different tools to assess them [47, 48], on cut-off-points, on clinical settings such as COPD stage and on clinical evolution, i.e. clinical stability in terms of absence of exacerbation [48, 49]. Since mental comorbidities are often not diagnosed [50], it is therefore important to consider depression as a modifiable factor in patients with COPD. Orlandi et al. also concluded that most studies typically analysed the symptoms of depression by questionnaires, without performing a clear professional diagnosis of depression in the studied patient groups [48, 51].

As the general level of activation in depressed patients is reduced [52], an explanation of the high significance of depression for results from the 6-minute walk test could be the lack of physical drive associated with depression. This means that these patients may lack the physical drive to walk as much as possible within 6 minutes. An anxious person, on the other hand, may still be motivated to walk as far as possible in this time frame.

Furthermore, the trend that depression is more associated with the progression of somatic diseases up to increased mortality than anxiety disorders can also be seen in studies with patients with cardiovascular disease [53–55]. Part of this observation could be due to the fact that depression is the more global construct of the two subscales.

Our study has important limitations. Firstly, it has a cross-sectional study design, which does not allow any clear causal conclusions to GOLD stages to be drawn. A longitudinal interventional format would be desirable to address the influence of GOLD stages. Secondly, our data cannot be generalized to all COPD patients, as only hospitalised patients in GOLD stages II to IV and having had an acute exacerbation, were recruited. Third, the 6-minute walk test was performed only once for each patient and thus represents an archetypical clinical situation. Repetitive intraindividual assessments would be preferable, also in order to be able to detect a possible causal link with time-series analyses, and mood and thus questionnaire results are also modified over time. Because of these limitations, the results of this study must be interpreted with caution.

In conclusion, the results of this study showed that psychological factors were associated with physical functional performance and therefore probably modulated physical functional performance in daily routine. It suggests that the relation between psychological factors and somatic measurements should be considered in clinical settings. At least part of them may be context-dependent. Indeed, COPD exacerbation leading to urgency hospitalisation represents a psychological stress associated with considerable mortality. Our study results may therefore differ from other, e.g. healthier study groups, or with other, e.g. clinically stable settings [48], or with much different 6-minute walking distances. Further studies should clarify the effects of factors leading to a reduction in depression, including medical and psychotherapeutic interventions [56], as well as physical activity, exercise and rehabilitation [57–60], which may also influence test results and may in parallel improve the quality of life and possibly the outcome of COPD patients [47].
